# Current Knowledge about Providing Drug–Drug Interaction Services for Patients—A Scoping Review

**DOI:** 10.3390/pharmacy9020069

**Published:** 2021-03-24

**Authors:** Tora Hammar, Sara Hamqvist, My Zetterholm, Päivi Jokela, Mexhid Ferati

**Affiliations:** 1 Department of Medicine and Optometry, The eHealth Institute, Linnaeus University, 391 82 Kalmar, Sweden; my.zetterholm@lnu.se; 2 Department of Media and Journalism, Linnaeus University, 391 82 Kalmar, Sweden; sara.hamqvist@lnu.se; 3 Department of Informatics, Linnaeus University, 391 82 Kalmar, Sweden; paivi.jokela@lnu.se (P.J.); mexhid.ferati@lnu.se (M.F.)

**Keywords:** drug–drug interaction, medication information, patient, patient empowerment, eHealth, digital medication management, clinical decision support system, design, usability, DDI-checker

## Abstract

Drug–drug interactions (DDIs) pose a major problem to patient safety. eHealth solutions have the potential to address this problem and generally improve medication management by providing digital services for health care professionals and patients. Clinical decision support systems (CDSS) to alert physicians or pharmacists about DDIs are common, and there is an extensive body of research about CDSS for professionals. Information about DDIs is commonly requested by patients, but little is known about providing similar support to patients. The aim of this scoping review was to explore and describe current knowledge about providing digital DDI services for patients. Using a broad search strategy and an established framework for scoping reviews, 19 papers were included. The results show that although some patients want to check for DDIs themselves, there are differences between patients, in terms of demands and ability. There are numerous DDI services available, but the existence of large variations regarding service quality implies potential safety issues. The review includes suggestions about design features but also indicates a substantial knowledge gap highlighting the need for further research about how to best design and provide digital DDI to patients without risking patient safety or having other unintended consequences.

## 1. Introduction

Pharmaceutical treatment is an essential part of health care and enables the cure and prevention of many conditions. However, drug-related problems (DRPs) are frequent and cause suffering for patients, and substantial costs for society [1,2,3,4,5]. DRPs are also a common reason for hospital care and they can sometimes be fatal [1,4,5,6,7,8]. A drug–drug interaction (DDI) can be described as the effect of one medication being changed (enhanced, diminished, or modified) by the presence of another medication when administered simultaneously or successively [9,10]. DDIs are common, with reported prevalence among hospitalized elderly patients ranging from 8 to 100%, and may compromise patient safety [11]. One of the key components to achieving appropriate drug treatment is the access to the needed information for the involved actors, such as prescribers, pharmacists, and patients [12,13,14]. Information and knowledge regarding medications are continuously increasing and changing as new treatments emerge, and as findings in research or clinical practice change existing recommendations [15].

eHealth solutions have the potential to address the problem of DDIs and generally improve medication management by providing digital services for health care professionals and patients. eHealth interventions will continue to transform many parts of medication management from consultation with health care professionals to learning about medicines and their management in daily life [16]. The management of DDIs is a complex process and it involves risk-benefit assessment of the involved drugs. Clinical Decision Support Systems (CDSSs) in the medication management process are used to improve health care quality and efficiency [7,10,11,12,13,14,15,16]. A CDSS can support health care professionals, such as physicians and pharmacists, in detecting potential DDIs by linking patient-specific factors and current medications together with a knowledge database. Developing a high quality knowledge database for DDIs requires extensive work and continuous updates and expert reviews [17]. Such a knowledge database can then be used in different applications and interfaces. Many studies have explored and evaluated CDSS that alert professionals about DDIs, by describing effects and aspects to consider when designing and implementing DDI alerts for professionals, mainly physicians [18,19,20]. However, less is known about providing DDI services for patients.

Patient empowerment is believed to facilitate patient independence, self-management, and self-efficacy [21]. For patients to feel safe and adhere to their medication treatment, it is of importance to provide understandable information that matches the individual patient’s information needs [22]. Besides the basic information needs (i.e., which medication to use, how, and when), patients may want more information to be able to evaluate the benefits of the prescribed drug and weigh this information against their concerns. If patients have a strong belief in benefits it may increase adherence. On the other hand, having a strong “concern belief” may instead lead to patients choosing not to take their medication (i.e., intentional non-adherence). It is believed that providing subjectively desired information about medications can prevent patients’ concerns. Although one of the most frequently requested topics regarding medications is DDI information, little is still known about how to customize this kind of information for patient needs [22]. Previous studies have shown a failure to meet the information needs of patients regarding DDI information and a gap between patient expectations and the information they receive from professionals [23,24].

Physicians and pharmacists, among others, have important roles in making sure patients’ treatments are safe and appropriate, for example, by avoiding DDIs. However, DDIs are still a large problem [25,26], indicating that they are sometimes missed by health care professionals. Reasons for missing a potential DDI may be a lack of knowledge about DDIs, a lack of appropriate CDSS in the information system they are using, or due to missing correct information about a patient’s current medications, or due to different perceptions about responsibility or lack of time to check for interactions [27,28,29]. DDIs can also occur because of the patient’s self-medication, such as Over the Counter (OTC) medications or herbal drugs that patients buy themselves, or because of medically unapproved re-use of prescription drugs or the consumption of dietary supplements [30]. There is a need to increase awareness among patients regarding DDIs, especially related to self-medication [30,31]. Written medication information to patients, such as a patient information leaflets (PIL) and online information sources, sometimes include DDI information [32]. There are some concerns among health care professionals—about providing DDI information to patients. The reasons include: perceived complexity of information; fear of creating patient anxiety, which could lead to patients not taking their medication; or increased concerns and unnecessary questions to health care professionals [33]. There are already some apps or services available for patients to check for DDI indicating an existing need or demand among patients [34,35,36,37,38]. However, there is limited knowledge about any effects or if their design or content is appropriate for patients.

It is still not clear how patients’ information needs, regarding DDIs, can be met while minimizing risks to patient safety or unwanted effects. In this scoping review, we describe current knowledge about providing patients with digital DDI services and checkers to identify knowledge gaps and areas for future research.

The aim of this scoping review is to explore and describe current knowledge about providing digital DDI services for patients. We focus on two research questions (RQ):

RQ1: what is known about patients’ needs, use, and understanding of DDI information, as well as any effects from providing it?

RQ2: what is known about design quality, content, and usability of interactive DDI services for patients (such as DDI checkers, apps, and websites)?

## 2. Materials and Methods

To address study RQs, in this scoping review, we used a methodical framework that was introduced by Arksey and O’Malley [39], which has been scrutinized and further developed by Levac et al. [40], Joanna Briggs Institute [41], and Daudt et al. [42]. The purpose of this study is to conduct a narrative review of a topic that has not previously been comprehensively reviewed and, as a result, provide an overview of the field as well as to identify research gaps in the existing literature. Moreover, the research team is multidisciplinary, which allows us to elucidate the subject area from different perspectives [40,42]. The joint knowledge of the team comprises subject areas of pharmacy, informatics, information design, public health, health informatics, and communication.

In the review, we utilize the framework that contains five stages plus an optional sixth stage, as follows [39]:

Stage 1: Identifying the research question;

Stage 2: Identifying relevant studies;

Stage 3: Study selection;

Stage 4: Charting the data;

Stage 5: Collating, summarizing and reporting the results;

Stage 6 (optional): consultation exercise (stakeholders and/or experts give feedback on review to validate results and add any missing pieces).

In this work, we used stages 1–5, but the optional sixth stage was not conducted. Our scoping study can also be considered as an iterative process, so that stage 1, as well as stages 2 and 3, were continuously informed by the review process. In this way, both research questions and eligibility criteria were gradually adjusted and refined during the scoping process.

### 2.1. Eligibility Criteria and Search Strategy

In this study, only peer-reviewed journals or conference papers written in English were included. Both research papers and review papers were eligible, but opinion papers were excluded. The search did not have a lower limit in the time period, so that with respect to time, all papers until January 2021 were eligible. In addition, the papers must focus on information of drug–drug interactions (DDI) that is aimed at patients. As defined in the research questions, the papers can describe patients’ needs, use, and understanding concerning the available DDI information, as well as the effects of providing the information to patients. The research questions also set forth that we included papers that concern the design quality, content and usability of interactive DDI services. Moreover, papers that described patient needs of DDI information more generally, and that did not focus on digital DDI services, could be included if the review authors deemed that these papers contained relevant knowledge about the review topic. Papers that only focused on DDI services for health care professionals were excluded, as well as papers that focused on oral communication about DDI between patients and health care professionals.

To identify relevant studies, we searched the following databases: ACM (Association for Computing Machinery), Google Scholar, IEEE (Institute of Electrical and Electronics Engineers), and PubMed. The search queries and results from different databases are shown in Table 1 and more details provided in Supplementary Text S1. In addition to the database search, the reference lists of the selected articles were searched using the same eligibility criteria as for the databases.

### 2.2. Selection and Analysis of Sources

The search for articles was conducted between October 2020 and January 2021. All authors participated in the initial screening, where more than 1000 papers were screened to find titles that may be relevant for the research questions (Table 1). From this initial collection, approximately 100 publications were selected for a second screening, where their abstracts were read. As we did not want to miss any significant contributions in this novel research field, the initial search terms were aimed to give a relatively wide range of papers, and the manual screening was then used to identify the papers that were relevant for our aim and the research questions. If the abstract did not provide enough information, the paper was read in full text to make the final decision of its relevance.

The authors analyzed the selected publications with a focus on the patient’s needs and the impact of DDI information (RQ1), as well as with focus on design and usability of interactive DDI services (RQ2). During the analysis, the selection and extraction of data were frequently discussed among the authors, until a consensus was reached concerning which papers should be included in the final review. Moreover, as was stated earlier, both research questions and eligibility criteria were gradually refined during the iterative scoping process.

## 3. Results

We found 19 papers meeting our inclusion criteria by being relevant for either RQ1, RQ2, or both, and these papers were included in the scoping review. An overview of the reviewed papers is shown in Table 2. The table includes: the lead author and publications year, study focus, and aim (what was studied), as well as the principal method (how the study was conducted) and geographical context (where the study was conducted). It is also indicated to which research question the paper is relevant for. In addition to the table, the key findings of the selected papers are described and summarized in text, separately for each of the research questions. Two papers had findings relevant for both RQ1 and RQ2, and are therefore included in both sections.

### 3.1. Key Findings of Papers Relevant to RQ1

In this review, 11 papers with results relevant for RQ1 were identified. They apply different methods and cover different perspectives related to patient needs, use, understanding, and any effects of using DDI information. Only one paper investigates real life experiences from patients using available digital DDI service. In the study by Justad et al. [50], a questionnaire was performed among patients who had used the Swedish DDI service Janusmed interactions, which is aimed at and designed for health care professionals. Patients described using the service to check for DDIs between prescribed medications, but also for OTC medication, herbal medication, food, and alcohol. They described different reasons for wanting to check DDIs themselves, including not trusting health care professionals to make sure there were no interactions. Many used it to check for DDIs for friends or family. The patients were asked how they would react if they found a DDI among other things. Although the majority answered that they would talk to the prescriber, some said they would consider adjusting the dosing or stop taking the drug depending on the circumstances and if the DDI involved OTC or prescribed medications.

Other papers investigate patients’ perspectives on DDI information, in general and in various settings. Four papers describe the need among patients for DDI information, using both qualitative and quantitative methods. Kusch et al. [22] reviewed the literature to describe drug information desired by patients, including 12 studies describing enquiries to drug information hotlines and 15 qualitative studies evaluating drug information needs. They found that the most frequently requested topic by patients was the information on adverse drug reactions (ADRs) and drug–drug interactions (DDIs). In this scoping review, we do not include the individual papers from the review by Kusch et al.

In a qualitative study involving older patients with multi-morbidity, Haverhals et al. [45] describe information needs regarding DDIs, as well as highlight major differences between individuals related to this. They found that many of the patients were worried about physicians prescribing medications without fully considering DDIs. Many patients used the package leaflet to look for side effects and interactions, and some actively searched for information online. Many wanted to remain independent, and to have a more participatory role in decision making, whereas others followed physician’s recommendations without question. They found that the patients in their study sometimes decided on their own to stop or alter their medications. However, fear of DDIs was not mentioned specifically in the paper as a reason for non-adherence, rather the reasons described included experiencing side effects or feeling that they were on too many medications. Sometimes they discussed this with their doctor, but others did it without consulting the doctor. Their results suggest that personal health applications for elderly to help in management of medications should include links to authoritative and reliable information on side effects and drug interactions among other things.

In a study assessing the patient’s information needs (regarding DDI), Mutebi et al. [53] conducted a questionnaire among registered users of an online medication monitoring service in order to inform future education resources. They found that most common concerns cited by the users were: the identification of interacting medications; seriousness of DDIs; interactions with OTC medications; interactions with foods; exacerbating comorbidities; short- and long-term adverse effects; signs and frequency of DDIs; and how to minimize adverse effects. The study showed that the gender, number of prescriptions, and the number of OTC medications were related to perceived importance of different types of DDI information.

The final paper, describing the need for DDI information, was the study by Indermitte et al. [49], about the patient’s knowledge regarding DDIs, with a focus on self-medication and OTC medication. Patients were often not aware of, or had too little knowledge about, DDIs. Moreover, they do not always tell prescribing doctors about the use of OTC and herbal medications. Since OTC drugs are not always bought at the same time as prescription medications, the authors concluded that it could potentially lead to serious consequences due to interactions with prescribed medications.

Among the papers included in this review, two studies focused on the patient’s comprehension and understanding of DDI information in patient information leaflets (PILs). Gustafsson et al. [44] examined and evaluated PILs for common medications using both experts and patients. Their study showed low comprehensibility among patients for the information items regarding DDIs and contraindications. In their study, they also describe reasons for poor comprehensibility including complexity of the messages, old age, and short formal education. Similar issues are described in a design study by Khodambashi et al. [51], in a sample of individuals with good computer skills, relatively high education and no medical background. The participants did not understand the concept of “drug interactions”; they had difficulties in understanding the information in PILS, and found them to be long, unstructured, and time-consuming to read. Some of them could recognize the drug interaction but did not understand the meaning of it.

In this review, no studies about effects of patients using digital DDI services (such as DDI checkers) were found. Four papers included in this review reported on the patient’s reactions or decisions related to DDI information in general. All of them used scenarios or experiments rather than actual real-life decisions or reactions. Herber et al. [46] used focus groups to explore patients’ reactions and related behaviors towards risk information presented in PILs and found that such information could lead to unplanned reactions. Information about side effects and drug interactions in PILs triggered emotional reactions and behavior among patients; fear was the emotional reaction that was most often mentioned; the behavioral responses mentioned the most by participants were to seek support and to stop taking the medication. Dohle et al. [43] did an experiment to test how the presentation of risk affects the patient’s precautions in a form of dosing behavior. They observed that providing information about increased probability of adverse effects from a DDI did lead to increased perceived risk and negative effect among participants, but did not make them adjust their dosing behavior. Dohle et al. concluded that people may struggle to transfer their knowledge of DDI risks into decision-making behaviors. In a qualitative study, Heringa et al. [47] examined aspects that influence patients’ preferences regarding DDI-information related to DDI-management options. They found that patients had limited knowledge regarding DDI and that their preferences were highly dependent on the provided information. They found that certain cognitive, emotional, personal, and situational aspects were connected to the preferences. In a similar study by Heringa et al. [48], they found that the preferences regarding DDI management differ among both pharmacists and patients. For example, some groups considered not having to switch medications as most important when choosing between different management options, while others considered curing the disease or avoiding extra blood sampling as having higher importance.

### 3.2. Key Findings of Papers Relevant to RQ2

After reviewing the literature, ten papers were found to be relevant for RQ2. Three papers contain a review conducted on App Store and Google Play to uncover apps that covered the feature of DDI and were aimed for patient use. A review study by Kim et al. reports on mHealth apps provided in English aimed at Canadian citizens to check for potential DDIs [36]. The review included 23 apps found on App Store and Google Play, which were evaluated for their comprehensiveness and accuracy in terms of DDIs. The results show that the majority of apps are of low quality and provide inaccurate and potentially unsafe information about DDIs. Specifically, using these 23 apps, they investigated 26 DDIs, of which only around 60% were identified and of which only less than 50% contained interactions that were correctly described. This means that only 30% of these apps could correctly identify and describe interactions. These weak results show that using these apps could be a serious threat to patient safety. The study also highlights that there was a high variability in terms of app quality. Half of the apps scored 4 out of 5 in terms of accuracy, whereas 30% of the apps scored only 1 out 5.

Similarly, Shiguang Loy et al. conducted a review of 59 apps, of which 40 apps included the DDI feature [37]. The results of the study indicate that app reliability was poor, although paid apps did provide a better usability. The assessment was done using a tool the authors developed that evaluates app usability. This tool, authors claim, could also be useful for app developers when designing DDI apps.

The final paper addressing mobile apps, Bailey et al., reports on a large sample assessment of mobile apps dedicated to patient self-management [35]. In total, they investigated 424 apps of which 12 apps contained the feature of DDI. The app evaluation process included an analysis of user reviews (*N* = 1091) found as a feedback on the store pages of these apps. Unfortunately, authors do not provide specific results only for apps DDI feature, but they claim that most apps contain common negative feedback such as crashing, freezing, and inconsistent performance. In addition, they discovered apps to have poor compatibility with certain medications.

Three papers, among the ten in total, describe DDI websites available for patient use. Adam and Vang investigated 44 such websites [34]. Some websites included in their study could only check for binary medication pairs, but the majority could assess three or more medications. These websites were evaluated for information capacity (clinical content), patient usability, and readability. In the evaluation, the researchers used five drug pairs with clinically significant DDI. In terms of information capacity and clinical content, websites were quantitatively rated in terms of: DDI alerts, severity grading, and its explanation. They found substantial variation in clinical content between the included websites, where some did not correctly identify clinically relevant DDIs. Additionally, the results indicate that the majority of websites lack severity rating, and there were variations in the rating among the sites that did include any severity ratings. They also found that several websites lacked data on therapeutic duplications, drug–food and drug–alcohol interactions. In terms of patient readability, they used a so-called Flesch–Kincaid grading model [56], which helps assessing whether the information provided is understandable. The results show that most of the investigated websites scored low in terms of readability, which is an indication that the provided information might be difficult for patients to interpret. In terms of patient usability, the average score for included websites was 2.9 (out 5) points. Only five websites received the highest usability score.

Similarly, the study by Vingen et al. reports on available DDI website checkers in Scandinavian market [38]. They identified three Norwegian (Interaksjoner.no, Felleskatalogen.no, Legemiddelsok.no), two Danish (Medicinkombination.dk and Interaktionsdatabasen.dk), and one Swedish (Janusmed) service. They conducted a heuristic evaluation using a qualitative and quantitative analysis. All (but one) checkers were primarily intended for use by professionals, but also being open for patient use. The results indicate that all checkers contain serious usability issues that could undermine patient user experience. In some cases, serious issues were identified by not clearly communicating a dangerous interaction between drugs. Additionally, checkers lacked adaptive web browsing design, especially for smartphone size screen. All checkers also lacked thesauri, which is important and useful as typically patients misspell drug names. Finally, the study indicates that all checkers failed to adhere to basic design principles, which could undermine patient usability.

The third study focuses on a Swedish website, which gives patients access to a DDI database aimed at health care professionals. In the study by Justad et al. [50], which included results relevant for both RQ1 and RQ2, patients using the DDI service were asked about perceived usability and comprehensiveness of texts and if they were lacking any information or functionality. Although the DDI service was designed for professionals, most patients answered that they found the database easy to use. However, the patients in that study seem to have high health literacy compared with patients in general, with several of them having some form of health-related education. Patients in general may perceive the DDI service more difficult to use. The patients also suggested some improvements and features, such as automatic connection with own medication lists and texts adapted for laymen.

The final four papers included in this research question describe prototype tools that authors have developed. The study conducted by Khodambashi et al. investigated the usability of a prototype they developed for DDI compared to PILs [51]. They developed the prototype using four workshops to help co-design the interface by involving patients and pharmacists. When evaluating the prototype against the PILs, they investigated the user performance in finding information, the level of their understanding of provided DDI information, and learning from the visualized information. Specifically, they collected the following data: task completion rate, task completion time, usability, and learnability. The results show that using the prototype, users were able to find the required information faster and they made fewer mistakes than when using PILs. They maintain that PILs typically contain more medical jargon and lack of personalized information for the user, which aspects they address in their prototype.

Similarly, the study by Spanakis et al. reports on a DDI tool they have developed for the purpose of patient empowerment [54]. The tool is aimed at helping patients to reduce cases of unwanted drug interactions. They have evaluated the tool with 35 patients during a two-month period to measure tool’s usability, reliability, and efficiency. The results indicate a positive attitude towards the tool. In a scale from 1 to 5, where 1 indicated low, whereas 5 indicated high, 30% of participants marked 4 and 59% marked 5 for the reliability; 18% marked 4 and 78% 5 for usability; and 27% marked 4 and 73% marked 5 for efficiency. The paper contains few details about how the evaluation was conducted. The same authors have also developed a mobile application called PharmActa, which, among many features, includes a DDI checker for patients. PharmActa is an eHealth platform under development to empower patients and provides a personalized pharmaceutical care [55]. With PharmActa, the plan is that patients will be able to access DDI checkers where they can check prescribed medications as well as adding OTC. The patient’s mobile app will then be connected with the pharmacist’s system to assist the pharmacist, to better review the patient’s medication and provide pharmaceutical care, enhancing the communication between pharmacists and patients. Using 33 health care professionals, they evaluated the application for usefulness, effectiveness, learnability, and satisfaction. The System Usability Scale (SUS) score for the DDI resulted in above 90, which indicates a high usability of the application by the users. Participants greatly appreciated the simplicity of the application and its visually pleasing interface. PharmActa was also perceived as highly effective enabling users to carry a fast and easy navigation through its available services. Participants required that an improvement to the application be done in order to enable users to edit the reminder settings. Another suggestion was to offer a medication pick list. PharmActa has not yet been evaluated among patients.

Finally, a study by Martin-Hammond et al. shows an iterative design process of a prototype developed to help people with OTC drugs [52]. Among other features, the prototype included a DDI service. They involved two experts and seven elderly users to design and evaluate the prototype in terms of likability, ease of use, language, information provided, etc. Participants preferred when the prototype offered a recommendation in addition to just information, when an interaction between drugs was found. For instance, they preferred the prototype to offer an alternative medication in cases when the drug they selected showed an interaction. Participants also viewed the prototype as a means to initiate better discussion with the doctor or pharmacist.

## 4. Discussion

This scoping review presents an overview of papers with findings related to the provision of DDI services to patients. The included papers describe a need among some patients for some form of DDI service or information and reasons for patients using digital DDI services. The papers also show that some patients have some difficulties understanding DDI information and that DDI information may lead to unplanned reactions. The studies describing the quality, content, and usability of available DDI services show issues and large variations regarding service quality, implying potential safety issues and difficulties for patients knowing what they can trust. Despite a broad search strategy, only a few papers studying DDI services for patients were found. This indicates a substantial knowledge gap. For example, no studies were found about effects of patients using digital DDI services in real life settings.

### 4.1. Patients’ Needs, Use, and Understanding of DDI Information, as Well as Any Effects

The need for DDI information is expressed in four of the included studies, by patients in various settings, and in studies using both qualitative and quantitative methods [22,45,49,53]. None of those studies describe the need specifically for digital DDI services, but digital DDI services, such as websites or apps with DDI checkers may be one way of meeting the need. From those studies, it is clear that the desire for this information differs between patients, implying the need for individualization. Some patients want to check their own (or a family member’s) medications for any potential DDIs, both for prescription medication and self-medication. Patients have different views concerning their own role in medication management. They also differ in their trust in health care professionals, where some patients trust that prescribers make sure their medications are safe to combine and others feel the need to check it themselves [45,50,57]. Other studies, not included in this review, highlight the need for DDI information to patients by demonstrating a gap between patient expectations and the information actually provided by professionals [24,33].

Although many patients ask for this information, there is hesitancy and controversy surrounding the topic of providing DDI services for patients. Concerns include—among other things—that this development may lead to unforeseen consequences, such as adherence issues, patients that suddenly discontinue their medication, or unnecessary contacts with health care. A Swedish study on pregnant women illustrate these concerns and the different perceptions among patients and professionals [58]. Most pregnant women found the knowledge database “Drugs and Birth Defects” (intended for professionals) valuable and easy to understand and felt that their anxiety decreased. Among the health care professionals, on the other hand, a larger proportion saw risks with patients reading the information in such publicly available resources.

Although no studies measure any real-life effects of patients using DDI services, there are indications from studies involving hypothetical DDI-scenarios that some concerns may be partly justified. Most patients say that they would consult their doctor, but a few might consider changing the treatment on their own [50]. There are also indications that DDI information can lead to fear or behavioral reactions [43,46]. These results indicate that DDI services should include recommendations about contacting health care professionals before making any changes.

Offering patients information about DDIs is not something new to digital DDI services. Patients already have access to and use information about DDI in PILs included in the medication package or provided from other sources. Two of the studies in this review describe that patients have some difficulties understanding DDI information in PILs. Problems with comprehension and understanding has been reported even in cases when a majority of patients have reported that leaflets are easy to read [59]. This is noteworthy, and highlights the need to test the readability of the information resources, as well as the comprehensibility among patients. This also indicates that subjective customer satisfaction approaches and self-assessment scales may not be sufficient to evaluate the quality and safety of different drug information models. Instead, there is a need for complimentary methods.

Other studies highlight the importance of patient involvement in the development of DDI-checkers. The patients’ preferences for information might vary due to the medical issue [60] and the user [61]. Consumer testing could be useful to make the information resources more patient-centered. One important aspect to consider is patient health literacy. Additionally, awareness of the variability of DDI management preferences is useful knowledge for shared decision making [48].

### 4.2. How to Design and Provide DDI Services for Patients

Interface design principles and guidelines for DDI interfaces could be obtained from several studies. Some of those suggestions come from studies specifically addressing DDI interfaces, whereas there are also studies that discuss electronic medical records and other medical interfaces, which practices also provide relevant guidance for DDI interfaces. Two papers included in this scoping review of the second research question, among other aspects, also provide suggestions on how DDI checkers should be designed for optimal usability. Starting from the issues they have uncovered, Adam and Vang start with suggestions on how to address negative aspects with the websites they reviewed [34]. They claim that the usability could be addressed by providing content that includes information on risk communication when two or more drugs interact. They suggest using percentage values in conjunction with symbols and color-coding to convey such information. In addition, the risk alerts should be communicated and prioritized by severity (clinical significance), should contain suitable justification, and also should offer available responses [62]. On this matter, Martin-Hammond et al. further suggest that it is insufficient to provide users just with medicine interaction information, but the interfaces should also guide the user with finding a solution [52]. However, the authors stress that when the interface provides a recommendation, it should be clearly communicated to the user that such recommendation does not replace professional advice. Implementing a support for sharing within the interface could help users to easily initiate a conversation with a pharmacist or a doctor [52]. In essence, the recommendation feature should be closely implemented with the sharing feature to avoid users becoming too reliant on the interface suggestions and consequently act without proper counselling.

Aspects of content readability within DDI interfaces are an important element, which could be improved by providing a simplified textual explanation when drug interactions and risks are found. A recommendation for this aspect includes limiting or avoiding medical terminology that patients are typically unfamiliar with [52,55]. Similar suggestions are found on other studies where a study by Eloy et al. suggests using short sentences comprised of up to ten words in order to accurately convey meanings [63]. In addition, this study suggests to also include simple multimedia information along with a textual content. On this matter, a study by Kasabwala et al. suggests that, in addition to maintaining caution with regards to the length of words, the choice of words is also important, thus suggesting using a layman terminology [64]. This study also suggests writing sentences using active voice, structured in bullets, when appropriate, and use supporting images and graphs. Similarly, the World Health Organization suggests that the language of the text should be at a sixth-grade readability level or below to increase information understandability [65]. From these studies, it is evident that providing the same content via different formats (text, image, video, etc.) increases its comprehension.

Studies also suggest the use of a medication pick list as a feature to help users recognize rather than recall often difficult to remember drug names [34,38,52,66]. Multiple-entry pick lists are preferred to offer user’s a possibility to select multiple drugs concurrently [55,62]. In addition, Vingen et al. also highlight the need to implement the feature of search completion suggestions, which would help users get suggestions by the interface as they start typing drug names. Such feature, however, should be carefully implemented to avoid generating unanticipated issues [67].

To address the issues with some drugs being written with different similar names, a thesauri should be embedded into the interface that will catch drug names, which may be written slightly differently, e.g., tyroksin, thyroxin, or thyroxin [38]. Finally, a study by Middleton et al. lists extensive design recommendations in the form of usability principles gathered from research, the industry, and clinical end-use studies that are relevant and could be utilized when designing DDI interfaces [68]. Adhering to established practices when designing interface objects is important to avoid user confusion [52]. Designers should consider all these aspects when designing DDI interfaces, but also, they should test their interfaces often involving target users to ensure high interface usability.

### 4.3. Other Aspects to Consider When DDI Services Are Provided to Patients

Besides the general usability aspects of DDI services, there are other aspects to consider if DDI services are provided to patients. One such thing is clinical content, such as clinical relevance of the alerts. Some of the studies included in RQ2 include these aspects in the assessment. The quality of a DDI service is inevitably dependent on the quality of the knowledge database. There are many different DDI databases differing in content and quality, such as significant differences in the ability to detect clinically relevant DDIs [34,69,70]. There are several benefits of having the same knowledge database for patients and professionals. The existence of several interfaces and applications using the same evidence-based content would be a great advantage to all actors in the health care system [58,71]. However, simply providing the same service to patients as to health care professionals seem to be a non-optimal approach even if some patients may appreciate it [34,50]. The information presented might require some medical knowledge and decisions often require complex risk-benefit assessment. A lack of medical knowledge is not only a problem for understanding the given information, but it is also important to draw conclusions about the things the patients do not get information about. Some knowledge databases for DDIs include only certain types of interactions, e.g., some include primarily pharmacokinetic interactions and not pharmacodynamic interactions, and there are variations if the database includes alerts about therapy duplications [34,72]. Some potentially dangerous combinations of medications, such as therapy duplication or pharmacodynamic interactions, may be apparent for professionals with medical training but not for patients. Consequently, if a patient believes that not getting an alert about potential DDIs when entering all their medications into such a service means they are safe to use together, when they are in fact not, could pose a risk to patient safety by giving a false sense of security [30,73]. Thus, when designing DDI services for patients, it is important to consider how to provide information about the content and limitations of the service to the user.

Studies on professionals’ use of DDI services could inform this area regarding design aspects and usability, even though there are some important differences to consider [74,75]. Some apps have been evaluated for health professionals, but could be available for patients [76]. Several of the evaluated apps seem to be used by patients, indicated by the findings by Justad et al. where patients described other sources they use to find information about DDIs [50].

Even if patients use a service that is connected to the same DDI knowledge database, they will not automatically know which potential DDIs that health care professionals have already considered. The prescriber may have made a conscious choice to prescribe two interacting medications based on the circumstances, by making dose adjustments or considering other factors. Future development in this area should explore possibilities of communicating this kind of information along with the information about prescribed medications, to decrease anxiety and unnecessary contacts with health care professionals. However, this is not a simple task, considering current challenges with interoperability in the medication management process. In the prototype described by Spanakis et al. [55], they are aiming at some form of shared platform for patients and health care professionals. One additional aspect to consider is the possible litigation between health care professionals and patients regarding DDIs, with possible judicial follow-up.

Because of the knowledge gap on this topic, it can be relevant to learn from related services and similar topics. One example is the topic of medication reminder apps for patients where there is a more extensive body of research on design, evaluation, and effects [77]. Results from those studies could advise parts of designing DDI services for patients. In addition, apps with DDI checkers are often combined with other functionalities such as reminders. What is more, in a proof of principle paper by Kusch et al. they developed a database format showing a method to structure ADR information in a way that facilitates customization [78], which may inform any work with DDI services. There is also some research about education to patients using digital tools that can increase the patient’s knowledge about DDIs [79,80]. Furthermore, research regarding risk communication and framing of information, specifically from the pharmaceutical area, could help inform any development of DDI information services [81,82].

Considering the facts that (1) there is a demand for this kind of service among some patients; (2) there are already several services available and possibly more to come with substantial variations in content and quality; (3) these services have complex nature and carry potentially serious consequences related to providing DDI services to patients; and finally (4) a knowledge gap exists regarding how to provide DDI services for patients without risking their safety or increasing unnecessary contacts with health care providers, further research to increase the knowledge is important. Additionally, national authorities and health care providers should consider their role in providing, assessing, or regulating DDI services for patients. Providing DDI services to patients raises questions about when, where, and to whom the patient should turn to get counselling related to the DDI-information delivered by the service. Moreover, it seems important to consider the aim with the service and the desired behavior. The available DDI services today have different providers, including private companies and health care or authorities [36,37,38].

### 4.4. Future Research—Importance of Research and Evaluation

There is a gap in knowledge regarding digital DDI services to patients. In line with the findings in the present review, a Cochrane review by Nicolson et al. concludes that there is a lack of studies evaluating internet-based medicines information to patients in general, making it a high priority in research to come [83]. Future research should study what information should be given to patients, and how, and the appropriate language and level of detail regarding management options, for example, if it should be similar to the information and reasoning provided to professionals or if it should only be an indication about contacting their physician or pharmacist.

If/when digital DDI services are implemented or provided, it is important to evaluate its use, perceptions, and any effects. Both positive and negative effects should be evaluated to identify any unintended consequence and indications of risk behavior, lowered adherence, or excessive contacts with health care. Different approaches for evaluation could be direct measurements of the use of the service, as well as patients’ self-reported use, behavior, and clinical outcomes. Results from the reviewed papers indicate that services with written DDI information and DDI checkers have mainly been used by active consumers with high health literacy. It is not clear what happens if or when it is promoted to the broader public. Other questions for future research could include how the relationship and responsibilities of patients and professionals could be affected. Future studies should also assess which groups are interested in using this kind of service, and which groups are actually using the services, in order to be able to address any inequalities.

Examples of aspects to study among a diverse collection of individuals from the target group is readability, comprehension rate, the users’ needs and how the user understands, relates to, uses, and reacts (emotionally and behaviorally) to such information. Interdisciplinary collaboration in future research should provide knowledge from different perspectives.

### 4.5. Method Discussion

As this was a scoping review, it was aimed to describe aspects that are covered in a wide range of papers, comprising a variety of methodologies and terminologies. In order to approach the research problem from various angles, it was beneficial that the literature search, as well as the selection and analysis of the papers, was conducted by a team of authors with different backgrounds. The methodological framework that we adopted for this study, combined with an iterative work process and frequent discussions within the team, enabled us to harness the potential of the multidisciplinary research group.

As was anticipated, the initial screening produced a large number of papers, so that no significant contributions would be overlooked. Therefore, the iterative scoping process was necessary to guide the gradual refinement of the research questions and eligibility criteria, and thus decrease the number of papers that were included in the final review.

It is pertinent to note that this scoping review reports and discusses the findings that are presented in the selected papers, but the review did not aim to assess the quality of the reviewed studies nor did we conduct any further analysis on the reported data. As this is not a systematic review, there may be relevant research that was missed. However, in many cases, the search processes that were conducted independently by different team members, resulted in the same papers, so that we may conclude that we have found the key publications in this novel research area.

## 5. Conclusions

This paper contributes with an overview of current knowledge regarding development and provision of digital DDI services for patients. In addition, it contributes with some suggestions about design features. From the literature it is clear that at least some patients want to check for DDIs themselves, regarding both prescribed medications and self-medication. Moreover, the review shows that there are numerous DDI services available, but also that there are large variations regarding service quality, which may imply potential safety issues and other concerns. It is not established how well the available DDI services meet the needs of patients, how they are used by patients, and what the effects (if any) are from patients using them. Results in this review indicate that although most patients reading DDI information would contact health care professionals before doing any changes, some might consider changing their medication on their own. DDI information and management are complex areas, and making an incorrect decision has the potential to lead to serious consequences. In addition, there are differences between patients, in terms of demands, literacy, and ability to use this kind of services.

One of the main findings of this review is the limited research about digital DDI services to patients, indicating a knowledge gap. For example, no studies were found about effects on patients when they are using digital DDI services in real life settings. Considering the multifaceted and complex nature of providing (or not providing) DDI services to patients, we also highlight the importance of interdisciplinary collaboration and further research about how to best design and provide digital DDI to patients without risking patient safety or having other unintended consequences.

## Figures and Tables

**Table 1:** Search queries used in the different databases and results.

Search Query	PubMed	ACM	IEEE	Google Scholar
#1 (“drug-drug interaction” AND patient)	1765	42	475	
#2 (patient OR consumer) AND (“drug interaction” OR “drug-drug interaction” OR DDI) AND information	893			
#3 (patient OR consumer) AND (“drug interaction” OR “drug-drug interaction” OR DDI) AND (“decision support” OR CDSS OR “DDI alert”)	149			
#4 (“drug interaction” OR “drug-drug interaction” OR DDI) AND “shared decision making”	5			
#5 (patient OR consumer) AND (“DDI checker”)	5			
#6 (“drug interaction” OR “drug-drug interaction” OR DDI) AND usability AND (“patient interface” OR “patient-oriented”)	1			185

**Table 2:** Overview of papers included in review in alphabetical order.

Author (Year) RQ	Focus (What)	Study Characteristics (How, Where)
Adam and Vang (2015) [34] RQ2	Quantitative evaluation of DDI websites intended for patient use	- Evaluation of 44 DDI websites - Quantitative evaluation - United States
Bailey et al. (2014) [35] RQ2	Assessment of apps intended for patient self-management	- Quantitative assessment of 424 apps in total, of which 12 included the DDI feature - United States
Dohle et al. (2017) [43] RQ1	Testing if providing individuals with information about a drug combination that presents a synergistic risk increase perception and influence dosing decisions	- Two experiments where patients were presented with scenarios providing different information about DDI - Adult participants (*n* = 565) - United States
Gustafsson et al. (2017) [44] RQ1	Evaluation of how well patients recognize and comprehend PIL, including information about DDIs, as well as reasons for poor comprehensibility	- PILs for 30 commonly prescribed medicines - PILs evaluated by experts using a protocol and patients using a questionnaire - Sweden
Haverhals et al. (2011) [45] RQ1	Elucidation of the medication self-management needs and strategies of older adults with multi-morbidity and their adult caregivers that can be addressed by an electronic personal health application	- Semi-structured interviews, individually and in exploratory/confirmatory focus groups - Purposive sample of 32 adult patients and 2 adult family caregivers - United States
Herber et al. (2014) [46] RQ1	Exploration of patients’ reactions and behavior towards risk information in PILs of commonly prescribed drugs by general practitioners	- Focus groups - Patients in general practitioners’ practices - Six focus groups with 35 patients - Germany
Heringa et al. (2018a) [47] RQ1	Exploration of aspects influencing patients’ preferences regarding DDI management	- Focus groups: patients in 5 different community pharmacies - Total of 38 participants, who have used cardiovascular drugs for over 1 year, distributed in five focus groups - Netherlands
Heringa et al. (2018b) [48] RQ1	Exploration of patients’ and pharmacists’ preferences regarding DDI management	- On-line questionnaires: choice-based conjoint task on a fictitious DDI - 178 pharmacists and 298 patients - Patients were older than 40 years, and all used cardiovascular drugs - Netherlands
Indermitte et al. (2007) [49] RQ1	Assessing prevalence and patient awareness of selected potential DDIs with self-medication	- Observation of 1183 pharmacy customers - Interview with 536 pharmacy customers - 14 community pharmacies - Switzerland
Justad et al. (2021) [50] RQ1 and RQ2	Evaluation of why and how patients use an online DDI service, how they perceive content and usability, and how they declare they would react if they found an interaction	- A web-based questionnaire among users who had registered as a “patient” (*n* = 406, response rate 12.6%) for a DDI service aimed at professionals - Sweden
Khodambashi et al. (2017) [51] RQ1 and RQ2	A comparative assessment of a prototype tool developed and evaluated for DDI	- A comparative mixed-methods evaluation between a developed prototype and PILs - Evaluation with 13 participants - Prototype co-designed with patients and pharmacists - Norway
Kim et al. (2018) [36] RQ2	Assessment of mHealth apps for DDIs found on App Store and Google Play aimed at Canadian citizens	- Quantitative assessment of 23 mHealth apps for information comprehensiveness and accuracy of DDI - Canada
Kusch et al. * (2018) [22] RQ1	Scoping review study that: - Describes drug information desired by patients - Analyzes how drug information can be customized to meet the patient’s individual needs	- 12 studies of patient enquiries to drug information hotlines and services - 15 qualitative studies evaluating drug information needs - Several countries
Martin-Hammond et al. (2015) [52] RQ2	Iterative design and evaluation of a prototype for OTC medication for patients, which among many features, includes a DDI service	- Design and evaluation of a prototype using two experts and seven elderly users - Qualitative study - United States
Mutebi et al. (2013) [53] RQ1	Assessment of patients’ information needs and preferences about potential DDIs, in order to inform patient DDI education resources	- On-line questionnaire - Sample of 100 registered users of an online medication monitoring service - United States
Shiguang Loy et al. (2016) [37] RQ2	Assessment of apps for DDI feature	- Quantitative assessment of 59 apps for DDI using a tool developed by authors - English apps without a geographically limited scope
Spanakis et al. (2016) [54] RQ2	Evaluation of a DDI tool developed for patient empowerment	- Quantitative evaluation of a DDI tool with 35 patients - Unspecified geographical scope
Spanakis et al. (2019) [55] RQ2	Evaluation and presentation of an eHealth platform including DDI checkers developed for patient empowerment	- Describing content of a platform and knowledge database - Pilot evaluation with 33 health professionals - Quantitative and qualitative evaluation - Greece
Vingen et al. (2020) [38] RQ2	Evaluation of usability issues that patients might face when using publicly available DDI checkers	- Mixed methods, heuristic expert evaluation - 6 websites evaluated on a mobile browser - Scandinavia

* The individual papers included in the review by Kusch et al. was not included in the present scoping review. RQ = research question; RQ1 = papers relevant for research question 1; RQ2 = findings relevant for research question 2; DDI = drug–drug interaction; PIL = patient information leaflet.

## Supplementary Materials

The following is available online at https://www.mdpi.com/2226-4787/9/2/69/s1, Supplementary Text S1: Search strategy for scoping review.
