# Supplementary material for: Current Knowledge about Providing Drug–Drug Interaction Services for Patients—A Scoping Review
Source: Pharmacy (Basel). 2021 Mar 24;9(2):69. doi: 10.3390/pharmacy9020069 (PMC8103271; doi:10.3390/pharmacy9020069)
Supplement: Supplementary file 1 [file pharmacy-09-00069-s001.pdf]

Supplementary Text S1: Search strategy for scoping review

Details from search in PubMed

#1
Search: **("drug-drug interaction" AND patient)** Filters: **from 1000/1/1 - 2021/1/31**
("drug-drug interaction"[All Fields] AND ("patient s"[All Fields] OR "patients"[MeSH Terms] OR "patients"[All Fields] OR "patient"[All Fields] OR "patients s"[All Fields])) AND (1000/1/1:2021/1/31[pdat])

#2
Search: **(patient OR consumer) AND ("drug interaction" OR "drug-drug interaction" OR DDI) AND information** Filters: **from 1000/1/1 - 2021/1/31**
(("patient s"[All Fields] OR "patients"[MeSH Terms] OR "patients"[All Fields] OR "patient"[All Fields] OR "patients s"[All Fields] OR ("consumable"[All Fields] OR "consumables"[All Fields] OR "consume"[All Fields] OR "consumed"[All Fields] OR "consumer"[All Fields] OR "consumer s"[All Fields] OR "consumers"[All Fields] OR "consumes"[All Fields] OR "consuming"[All Fields])) AND ("drug interaction"[All Fields] OR "drug-drug interaction"[All Fields] OR ("dig dis interv"[Journal] OR "ddi"[All Fields])) AND ("inform"[All Fields] OR "informal"[All Fields] OR "informality"[All Fields] OR "informally"[All Fields] OR "informant"[All Fields] OR "informant s"[All Fields] OR "informants"[All Fields] OR "information"[All Fields] OR "information s"[All Fields] OR "informational"[All Fields] OR "informations"[All Fields] OR "informative"[All Fields] OR "informatively"[All Fields] OR "informativeness"[All Fields] OR "informativity"[All Fields] OR "informed"[All Fields] OR "informer"[All Fields] OR "informers"[All Fields] OR "informing"[All Fields] OR "informs"[All Fields])) AND (1000/1/1:2021/1/31[pdat])

#3
Search: **(patient OR consumer) AND ("drug interaction" OR "drug-drug interaction" OR DDI) AND ("decision support" OR CDSS OR "DDI alert")** Filters: **from 1000/1/1 - 2021/1/31**
(("patient s"[All Fields] OR "patients"[MeSH Terms] OR "patients"[All Fields] OR "patient"[All Fields] OR "patients s"[All Fields] OR ("consumable"[All Fields] OR "consumables"[All Fields] OR "consume"[All Fields] OR "consumed"[All Fields] OR "consumer"[All Fields] OR "consumer s"[All Fields] OR "consumers"[All Fields] OR "consumes"[All Fields] OR "consuming"[All Fields])) AND ("drug interaction"[All Fields] OR "drug-drug interaction"[All Fields] OR ("dig dis interv"[Journal] OR "ddi"[All Fields])) AND ("decision support"[All Fields] OR "CDSS"[All Fields] OR "DDI alert"[All Fields])) AND (1000/1/1:2021/1/31[pdat])

#4
Search: **("drug interaction" OR "drug-drug interaction" OR DDI) AND "shared decision making"**
(("drug interaction"[All Fields] OR "drug-drug interaction"[All Fields] OR ("dig dis interv"[Journal] OR "ddi"[All Fields])) AND "shared decision making"[All Fields]) AND (1000/1/1:2021/1/31[pdat])

#5
Search: **(patient OR consumer) AND ("DDI checker")**
(("patient s"[All Fields] OR "patients"[MeSH Terms] OR "patients"[All Fields] OR "patient"[All Fields] OR "patients s"[All Fields] OR ("consumable"[All Fields] OR "consumables"[All Fields] OR "consume"[All Fields] OR "consumed"[All Fields] OR "consumer"[All Fields] OR "consumer s"[All Fields] OR "consumers"[All Fields] OR "consumes"[All Fields] OR "consuming"[All Fields])) AND "DDI checker"[All Fields]) AND (1000/1/1:2021/1/31[pdat])

Details from search in ACM

#1
ACM DL: Query Name,Search Result Count,Query Syntax

PDT,42,"query": {AllField:("drug-drug interaction") AND AllField:(patient)} "filter": {Publication Date: (01/01/1908 TO 01/31/2021)},{NOT VirtualContent: true},{ACM Content: DL}

Details from search in IEEE

#1
("Full Text & Metadata":“drug-drug interaction” AND "Full Text & Metadata":patient)
